# Coming out of the Integrative Oncology Comfort Zone: Addressing Healthcare Providers' Wartime‐Related Concerns

**DOI:** 10.1002/pon.70042

**Published:** 2024-12-15

**Authors:** Shir Eliyas, Orit Gressel, Eran Ben‐Arye, Jan Vagedes, Noah Samuels, Sameer Kassem

**Affiliations:** ^1^ Rappaport Faculty of Medicine Technion‐Israel Institute of Technology Haifa Israel; ^2^ Integrative Oncology Program The Oncology Service, Lin, Zebulun, and Carmel Medical Centers Clalit Health Services Haifa Israel; ^3^ ARCIM Institute Filderstadt Germany; ^4^ Department of Neonatology University Hospital Tuebingen Tubingen Germany; ^5^ Center for Integrative Complementary Medicine Shaare Zedek Medical Center Faculty of Medicine Hebrew University of Jerusalem Jerusalem Israel; ^6^ Internal Medicine Department Carmel Medical Center Haifa Israel

**Keywords:** anxiety, healthcare providers, integrative medicine, integrative oncology practitioners, quality of life, resilience, war

## Abstract

**Study objective:**

To assess the impact of a personalized integrative medicine (IM) intervention on healthcare providers (HCPs) expressing war‐related emotional/spiritual and physical concerns.

**Methods:**

Physicians, nurses, para‐medical and other HCPs from 5 hospital departments in northern Israel underwent IM treatments provided by IM‐trained practitioners working in integrative oncology (IO) care settings. The two main HCP‐reported concerns were scored (from 0 to 6) before and following the intervention using the Measure Yourself Concerns and Wellbeing questionnaire. Post‐intervention narratives were examined for emotional/spiritual keywords (ESKs).

**Results:**

Of 190 participating HCPs, 121 (63.7%) expressed ESKs in post‐treatment narratives (ESK group), with 69 not expressing ESKs (nESK group). Both groups had similar demographic and professional backgrounds, and reported improved measure yourself concerns and well‐being (MYCAW) QoL‐related concerns immediately post‐intervention. However, between‐group analysis found significantly greater improvement in the ESK group for the first (*p* < 0.001) and second (*p* = 0.01) MYCAW concerns, as well as emotional/spiritual concerns (*p* < 0.001). Pain‐related concerns improved similarly in both groups, with improved scores continuing to 24‐h post‐treatment.

**Conclusions:**

HCPs with war‐related emotional/spiritual and physical QoL‐related concerns showed significant improvement following the IM intervention. This was more significant among those reporting ESKs for their two major and emotional/spiritual concerns, with pain scores improving similarly in both groups. Future research needs to explore specific and non‐specific effects of IM intervention provided by IO practitioners working outside their “comfort zone”, fostering collaboration between IM and mental health providers to address HCP wellbeing and resilience during a time of national crisis.

## 1 Background

Healthcare providers (HCPs) invariably face stressors such as overwhelming workloads; organizational confrontations; and clinical, communication and ethical‐related challenges. At the same time, they are expected to contend with the emotional/spiritual toll of caring for patients' suffering and disability. A hospital‐based study conducted during the COVID‐19 pandemic highlighted the vulnerabilities of HCPs to stressors, including compassion fatigue, burnout, and secondary traumatic stress [1]. Despite mental the health resources and services provided during the pandemic [2], a qualitative meta‐synthesis identified a number of barriers, with unaddressed needs [3]. Integrative Medicine (IM) therapies, including acupuncture, mind‐body and touch‐movement therapies, were provided to frontline HCPs working in isolated COVID‐19 departments, supporting well‐being [4]. IM‐treated HCPs reported improved emotional/spiritual and physical concerns and well‐being [5].

During wartime, HCPs have demonstrated significant mental health morbidity, with increased risk for anxiety, depression, and post‐traumatic stress disorder (PTSD) [6]. During the current war in Israel, 84% of hospital‐based HCPs in southern Israel reported feeling “insecure” on their way to work [7]. A study of Israeli mental health workers conducted a month after the beginning of the war identified a spectrum of adverse and adaptive reactions [8]. In a study exploring emotional coping among HCPs from October 2023, Levaot and Dolfin found organizational efforts helped mitigate potentially negative effects from exposure to traumatic stress [9]. The present study explored the impact of an IM intervention, provided by practitioners working in an integrative oncology (IO) service, on HCPs expressing war‐related physical and emotional/spiritual concerns.

## 2 Materials & Methods

### 2.1 Study Design and Participants

This prospective, non‐randomized, and non‐controlled study took place at the Carmel Medical Center in Haifa, Israel, from October 23, 2023 to February 2, 2024. HCPs and non‐clinical personnel aged ≥ 18 years working in one of five in‐patient hospital departments (internal medicine, surgery, emergency medicine, gynecology and obstetrics, intensive care) at the study center were eligible for inclusion. These included physicians, nurses, paramedical practitioners (e.g., physiotherapists, occupational therapists), medical technicians (e.g., respiratory therapists, x‐ray technicians), administrative and cleaning personnel.

### 2.2 Study Objectives

The primary study objective was to assess the immediate effect of the IM intervention on war‐related QoL concerns. Secondary study objectives included:

- Exploring specific war‐related QoL concerns associated with the war.
- Determining whether improved QoL scores were associated with expression of emotional‐spiritual keywords (ESKs).
- Assessing the duration of the effect of the IM intervention after 24 h.

### 2.3 Integrative Medicine Intervention

Eligible HCPs reporting war‐related QoL concerns were referred by hospital administration, senior physicians and nurses to IM practitioners working in IO services at the Lin and Carmel Medical centers in Haifa, Israel. These practitioners completed 10 h of acute stress disorder (ASD)‐focused training, co‐designed by psychologists and IM physicians [10]. Treatments were provided during working hours in designated rooms in internal medicine and emergency departments, lasting 30 min, with an additional 10 min for pre‐ and post‐treatment assessment. Treatments were individualized, addressing QoL‐related concerns, with at least one of the following: acupuncture, manual‐movement (e.g., acupressure, reflexology), and mind‐body modalities (e.g., relaxation techniques). Treatments took place with participants in a supine position and focusing on relaxed breathing.

### 2.4 Assessment of HCPs QoL‐Related Concerns

HCPs provided personal (age, gender, family status, religion and religiosity) and professional information (profession, years of experience, hospital department); previous experience with complementary medicine and mental health services. QoL‐related concerns were assessed using the measure yourself concerns and well‐being (MYCAW) questionnaire, asking participants to list and score their two most significant concerns and overall well‐being (from 0, not bothering me at all; to 6, bothers me greatly), immediately before and at 24 h post‐intervention [11]. MYCAW‐reported pain and emotional/spiritual concerns (e.g., anxiety, despair, sadness, frustration, stress) were designated as MYCAW pain or emotional/spiritual‐related concerns.

At post‐treatment, HCPs re‐scored the two MYCAW concerns and well‐being, then answering two open‐ended questions about the IM intervention. Free‐text narratives were analyzed using qualitative research methodology and ATLAS.ti Scientific Software (V.8) to enable systematic coding [12]. Post‐treatment narratives expressing ESKs relating to “calmness,” “relaxation,” “tranquility,” “levity,” “release,” and “disengagement” were analyzed in accordance with a previously used approach [13].

### 2.5 Statistical Analysis

Statistical analyses were conducted using R (version 4.4.1) and RStudio (version 2024.04.2.764). Descriptive statistics for baseline values were presented within each group. For binary and categorical variables, both absolute and relative frequencies were included. For continuous variables, means and standard deviations (SDs) as well as medians were used. Baseline *p*‐values were calculated using a chi‐square test for categorical data, or *t*‐test for continuous data.

The primary study outcome (impact of IM on two MYCAW concerns) compared pre‐ and immediate post‐assessment changes between ESKs and nESKs groups using an ANCOVA, adjusting for baseline (T0) measurements of the first MYCAW concern and well‐being as covariates. Secondary outcomes analyzed included MYCAW first and second concerns; well‐being scores; MYCAW pain and emotional/spiritual scores, all at baseline, immediately post‐treatment and within 24–48 h post‐treatment. Outcomes were reported as mean ± SD and median (MAD). Missing values were imputed using Multivariate Imputation by Chained Equations (MICE), with pooled statistics analyzed to perform sensitivity analyses. All statistical tests were two‐tailed, with significance levels set at 5% and confidence intervals at 95%.

### 2.6 Ethical Considerations

The Carmel Medical Center Institutional Review Board exempted the study from requiring written consent (exemption date October 15, 2023), and the study was registered at ClinicalTrials.gov (NCT06612749). Participation was voluntary, with no financial or other incentives offered.

## 3 Results

### 3.1 Characteristics of the Study Group

Of 190 participating HCPs, 121 (63.7%) expressed ESKs (ESK group) and 69 did not (nESK group). Both groups had similar demographic data, including age, gender, religion (Jewish/other), religiosity (secular, traditional, orthodox), family status (married/not); professional background (physician, nurse, para‐medical, other) and department (internal medicine, surgical, emergency room, administrative, other); and QoL‐related concerns (emotional, pain, insomnia, gastrointestinal, respiratory, fatigue). Baseline MYCAW emotional/spiritual scores (53.3%) and pain (32.3%) were reported with similar frequency in both groups; well‐being scores more frequently in the ESK group (*p* = 0.028).

### 3.2 Integrative Medicine Modalities

IM modalities were provided alone or as a multi‐modal regimen, including touch‐movement therapies (94.7%, 180 participants), acupuncture (45.3%, 86), and mind‐body therapies (37.4%, 71). Additionally, 14.7% (28) received instructions for self‐treatment at home.

### 3.3 Changes in QoL‐Related Concerns

Table 1 compares the response of the two groups to the IM intervention. The ESK group (vs. nESK) reported significantly greater improvement in baseline‐to‐immediate post‐intervention MYCAW (first, *p* < 0.001; second, *p* = 0.010) and emotional/spiritual (*p* < 0.0001) concerns. After adjusting for baseline first concern and well‐being scores, a statistically significant between‐group difference was shown for first concern scores immediately post‐intervention (F(3, 184) = 17.01, *p* < 0.001). Post‐Hoc for ESKs (mean_adj_ = 1.87; SE_adj_ = 0.13) had significantly lower values than nESKs (mean_adj_ = 2.69; SE_adj_ = 0.17) after adjustment (ΔMean_adj_ = 0.80; 95% CI_adj_: 0.38, 1.22; P‐adj <0.001). Within‐group baseline‐to‐immediate post‐intervention improvement was significant in both groups for MYCAW pain and QoL‐related concerns.

**TABLE 1:** Impact of the integrative medicine (IM) program before/after the treatment: HCPs expressing versus not expressing emotional‐spiritual keywords (ESKs).

Parameter	Pre‐treatment assessment	Post‐treatment assessment	Pre‐treatment assessment	Post‐treatment assessment	*p* values^a^
	Mean score ± SD (median)		Mean score ± SD (median)		
	HCPs reporting MYCAW^a^ concerns during the IM session				
	Expressing ESKs		Not expressing ESKs		
First MYCAW concern scores^b^	*N* = 121	*N* = 121	*N* = 68	*N* = 67	*P* ^1^ = 0.907; *P* ^2 ^< 0.001 *P* ^3^ < 0.001; *P* ^4^ < 0.001
	4.58 ± 0.99	1.88 ± 1.51	4.56 ± 1.18	2.67 ± 1.55	
	5	2	5	3	
Second MYCAW concern scores^b^	*N* = 100	*N* = 97	*N* = 58	*N* = 58	*P* ^1^ = 0.950; *P* ^2 ^< 0.001 *P* ^3^ < 0.001; *P* ^4^ = 0.010
	4.35 ± 1.26	1.93 ± 1.46	4.36 ± 1.12	2.60 ± 1.44	
	4	2	4	3	
Emotional concerns score^c^	*N* = 122	*N* = 122	*N* = 63	*N* = 63	*P* ^1^ = 0.245; *P* ^2^ < 0.001 *P* ^3^ < 0.001; *P* ^4^ < 0.001
	4.70 ± 1.02	1.78 ± 1.38	4.51 ± 1.12	2.49 ± 1.57	
	5	2	5	2	
Pain concerns score^c^	*N* = 69	*N* = 69	*N* = 43	*N* = 43	*P* ^1^ = 0.201; *P* ^2^ < 0.001 *P* ^3^ < 0.001; P^4^ = 0.596
	4.12 ± 1.19	1.97 ± 1.51	4.42 ± 1.22	2.42 ± 1.33	
	4	2	4	2	
Well‐being score^b^	*N* = 121	*N* = 121	*N* = 69	*N* = 68	*P* ^1^ = 0.028; *P* ^2^ < 0.001 *P* ^3^ < 0.001; *P* ^4^ = 0.003
	2.67 ± 1.31	1.29 ± 1.11	2.14 ± 1.68	1.34 ± 1.11	
	3	1	2	1	

*Note: n* = number of MYCAW concerns reported by HCPs.

^a^ MYCAW, Measure Yourself Concerns and Well‐being questionnaire scores the two most significant concerns ranging from 0 (not bothering me at all) to 6 (bothers me greatly).

^b^ Refers to the number of patients who expressed distress.

^c^ Refers to the number of distresses expressed by the treated staff members.

*p* values are presented concerning the following comparisons between the groups: *P* ^1^ = compared ESKs‐expressed to non‐ESKs‐expressed group baseline scores; *P* ^2^ = within ESKs‐expressed group score changes from baseline to post‐IM treatment assessment; *P* ^3^ = within non ESKs‐expressed group score changes from baseline to post‐IM treatment assessment; *P* ^4^ = between ESKs‐expressed to non‐ESKs‐expressed group changes from baseline to post‐IM treatment assessment.

### 3.4 Assessment at 24‐h Post‐Treatment

**FIGURE 1:**
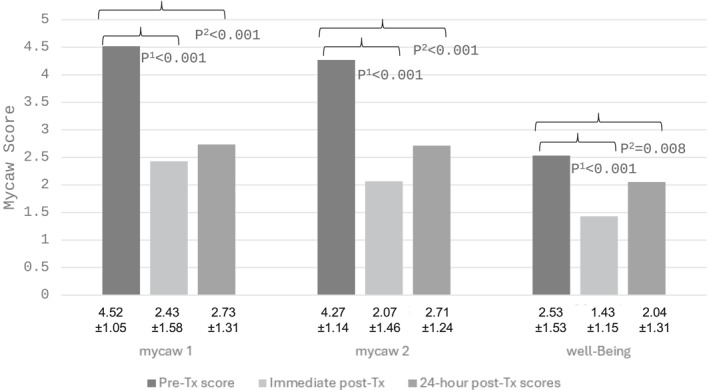
Change in HCPs concerns and wellbeing on MYCAW scores comparing pre‐to immediate and 24‐hour post‐assessments. P^1^ = changes in MYCAW scores pre to post treatment; P^2^ = changes in MYCAW scores pre to 24h‐post treatment. HCPs, healthcare providers; MYCAW, measure yourself concerns and well‐being; Tx, treatment.

Of 190 HCPs assessed at baseline, 79 (41%) completed 24‐hour post‐treatment questionnaires, with no difference between groups. Statistically significant reductions in MYCAW concerns, assessed immediately post‐treatment, persisted at 24‐h for both groups (*p* < 0.001). MYCAW well‐being improvement persisted from immediate‐to 24‐hour post‐treatment assessments (*p* = 0.008) (Figure 1).

### 3.5 Safety‐Related Issues

Adverse events associated with IM treatments were infrequent and mild. These included local discomfort during acupuncture; and difficulty relaxing at the beginning of mind‐body interventions, which resolved shortly thereafter.

## 4 Discussion

This study explored the impact of an IM intervention on three aspects of HCP QoL concerns attributed to the current war in Israel: Identifying war‐related effects on QoL and wellbeing among HCPs who, prior to the current conflict, were exposed to professional and personal challenges; creating a setting providing IM therapies addressing war‐related concerns in the hospital setting during work hours; and the provision of IM treatments by practitioners working in IO services, who received 10 h of acute stress disorder (ASD)‐focused training, co‐designed by psychologists and IM physicians. The impact of the intervention was assessed using both quantitative (MYCAW scores) and qualitative (MYCAW narratives) methodologies, comparing HCPs reporting ESKs to those who did not.

A significant improvement in HCP‐reported concerns was observed in both groups post‐intervention, persisting to 24 h post‐treatment. While between‐group analysis indicated that the ESK group improved more significantly for the two MYCAW and emotional/spiritual concerns, both groups showed similar improvement for pain and wellbeing, though the ESK group had significantly more severe baseline scores. The association between expressing ESKs and response to IM treatments was explored in an isolated COVID‐19 department in Israel [14]. There, nESK HCPs improved more significantly on the two MYCAW and emotional/spiritual concerns [5]. Objective measures, including heart rate variability, found increased parasympathetic activity, primarily among HCPs expressing ESKs, with decreased sympathetic tone in the nESK group [15]. This differs from the present study, possibly due to different “stressor stimuli” resulting from war‐related emotional/spiritual stress, which was not present during the COVID‐19 pandemic. The COVID‐19 study also found nurses and other HCPs reporting increased vulnerability to both mental and physical exhaustion, as well as physical pain [16, 17].

It is unclear why emotional/spiritual concerns improved more significantly from baseline to immediate post‐treatment in the ESK group, while pain improved similarly in both groups. Future qualitative research needs to explore the relationship between pain and emotional/spiritual concerns to understand whether HCP‐expressed pain reflects a somatization of deep emotional/spiritual and unspoken concerns; or a more “physical” form of pain reflecting exhaustion and “bio‐physical” concerns. The greater effect of IM on emotional/spiritual concerns may have been related to the ASD training that the IO practitioners received, helping them better address these concerns.

### 4.1 Clinical Implications

The study's findings support a beneficial role for IM in treating HCPs reporting war‐related emotional/spiritual and physical concerns, providing relief for these and other QoL‐related concerns, for at least 24 h. The two MYCAW symptoms and emotional/spiritual concerns responded more significantly among HCPs expressing ESKs, with equivocal benefit for pain. It is possible that during times of crisis HCPs who are less verbally expressive of ESKs may still benefit from the IM intervention. Further research should explore the role IM can play in preventing and promoting resilience among HCPs working in stressful settings. These programs should be co‐designed with mental health providers, with training provided to IM practitioners working in settings with intense emotional/spiritual stressors, such as integrative and psycho‐oncology, which may be outside their “comfort zone”.

### 4.2 Study Limitations

The study methodology is limited, primarily by the lack of randomization or control intervention. The comparison based on expressing ESKs in post‐intervention (vs. pre‐intervention) narratives precludes reaching any firm conclusions, though both study groups had similar baseline characteristics. In contrast to the use of uniform treatment regimens, the study's use of personalized IM treatments tailored to preferences and QoL‐related concerns is less rigorous. A potential selection bias regarding the referral of HCPs to the IM program may have existed, considering the relatively vague indication of reporting war‐related concerns. HCPs referred to the program may have had less significant war‐related concerns; while others not referred may have been reluctant to share their war‐related concerns and/or experience with IM practitioners, unfamiliar or skeptical to these practices. Long‐term assessment on the impact of IM in preventing burnout and psychiatric morbidity among HCPs working in highly stressful environments is needed. Finally, the study took place in a single medical center in northern Israel, limiting the generalizability of the findings to other settings in Israel or other countries experiencing similar or other forms of crisis.

## 5 Conclusion

The present study suggests that an IM intervention can impact HCP war‐related emotional/spiritual and physical concerns during the current conflict in Israel. While these effects were more significant among HCPs expressing ESKs, they were also significant for those not expressing ESKs, particularly regarding pain‐related concerns. Future research needs to explore the extent of non‐specific and specific effects of the intervention, using subjective HCP‐derived narratives and objective stress‐related parameters. Collaborative programs should be co‐designed by IM and mental health providers with an integrative approach to improve HCP wellbeing and resilience during this national crisis.

## Author Contributions

S.E., O.G., E.B.A., J.V., N.S., and S.K. organized the trial and collected the data analyzed this study. E.B.A., O.G., and S.K. planned the study. S.E., O.G., E.B.A., J.V., and S.K. carried out the analysis and wrote a draft manuscript. All authors participated in the revision of the manuscript, given final approval of the version to be published, and agreed to be accountable for all aspects of the work in ensuring that questions related to the accuracy or integrity of any part of the work are appropriately investigated and resolved.

## Ethics Statement

After reviewing the study protocol, the Carmel Medical Center Institutional Review Board (IRB) exempted the study from the need to obtain written consent. Following, the study was registered at ClinicalTrials.gov (NCT06612749).

## Consent

The study participants provided written informed consent.

## Conflicts of Interest

All authors declare that they have received no support from any organization for the submitted work; have no financial relationships with any organizations that might have an interest in the submitted work during the previous 3 years; and have no other relationships or activities that could appear to have influenced the submitted work.

## Data Availability

The data that support the findings of this study are available on request from the corresponding author. The data are not publicly available due to privacy or ethical restrictions.
